# Evaluation of the Optimal Protein Required in Granulated Microdiets for Rockfish (*Sebastes schlegeli*) Larvae

**DOI:** 10.1155/2022/2270384

**Published:** 2022-08-29

**Authors:** Bok Il Jang, Olumide Samuel Olowe, Sung Hwoan Cho

**Affiliations:** ^1^Chunhajeil Feed, Haman-gun, Gyeongsangnam-do, Republic of Korea; ^2^Department of Convergence Education of Maritime & Ocean Culture-Contents, Korea Maritime and Ocean University, Busan 49112, Republic of Korea; ^3^Division of Marine Bioscience, Korea Maritime and Ocean University, Busan 49112, Republic of Korea

## Abstract

Protein is an essential nutrient that supports fish growth, and the inadequacy in formulating their diets with an optimum protein level can deteriorate their growth performance. The protein requirement in granulated microdiets was estimated for rockfish (*Sebastes schlegeli*) larvae. Five granulated microdiets (CP42, CP46, CP50, CP54, and CP58) containing 42% to 58% crude protein levels with a 4% increment at a constant gross energy level (18.4 kJ/g diets) were prepared. The formulated microdiets were also compared with imported microdiets, Inve (IV) and love larva (LL) from Belgium and Japan, respectively, and a locally marketed feed (crumble). At the cessation of the study, the survival of larval fish was not different (*P* > 0.05), but the weight gain (%) of fish fed the CP54, IV, and LL diets was significantly (*P* < 0.0001) higher than that of larval fish fed the CP58, CP50, CP46, and CP42 diets. The crumble diet achieved the poorest weight gain of larval fish. Furthermore, the total length of rockfish larvae fed the IV and LL diets was significantly (*P* < 0.0001) longer than that of the fish fed all other diets. The chemical composition of the fish's whole body, except for ash content, was not influenced by the experimental diets. The experimental diets affected essential amino acid profiles, such as histidine, leucine, and threonine, and nonessential amino acid profiles, such as alanine, glutamic acid, and proline of the whole body of larval fish. Conclusively, based on the broken line analysis of weight gain of larval rockfish, protein requirement in granulated microdiets was estimated to be 54.0%.

## 1. Introduction

Rockfish (*Sebastes schlegeli*) is one of the main finfish farmed in the Republic of Korea (hereafter, Korea). The quantity of this marine fish species was 20,348 metric tons in 2019 with a value of approximately $133.4 million [1]. This fish was adopted in Korea due to its hardiness, fast-growing nature, and ability to grow at low temperatures [2]. Marine fish larvae are susceptible to nutritional challenges in the early developmental stages [3], and this is also applicable to rockfish larvae. A bottleneck to the production of rockfish is its low survivability at the larval stages, which can be attributed to feeding [2]. The fish larvae first feeding is important because it determines the effectiveness of fish feeds on the larval growth and production. After all, their development depends on how they absorb nutrients from feeds [4]. During the early days of development, live food is required for marine fishes [5], and in the case of rockfish, rotifer (*Brachionus plicatilis*) and *Artemia* nauplii are the main live foods at the larval stages. Although live foods are easy to consume and digest, they are likely to be deficient in some nutrients [2, 6, 7]. Therefore, it is necessary to develop formulated microdiets with optimum amino acid contents that could reduce the prolonged use of live foods and improve larval survival and growth.

The understanding of larval growth based on nutrient requirements is imperative, yet there is a paucity of knowledge on the nutrient requirements [8, 9]. Microdiets are formulated for fish larvae to improve their growth through adequate provision of nutrients that could enhance growth, increase hatchery efficiency, and reduce the cost associated with the prolonged use of live food organisms [9–11]. However, the size of the fish mouth, gut capacity, acceptance, and digestibility of microdiets are bottlenecks for larval culture [5, 6, 12]. Generally, microdiets are administered after the larval fish are reared with live food [11] or in combination with live food. The combination of *Moina* and microdiet achieved better growth performance for Ripon barbel (*Barbus altianalis*) larvae (48 days after hatching) than other diets (decapsulated *Artemia*, *Artemia* nauplii, Moina, or dry feed) [13]. The early use of microdiets improved the growth and reproductive performance of zebrafish (*Danio rerio*) [14], as well as the survival and growth of Southern flounder (*Paralichthys lethostigma*) larvae when fed with formulated microdiets consisting of a mixture of marine protein sources and attractants [10]. Additionally, the use of granulated microdiets in olive flounder (*Paralichthys olivaceus*) larvae improved their growth performance [15]. These studies used high protein ingredients, suggesting the importance of protein to the effectiveness of microdiets in larval fish. A low protein level in microdiets might cause poor performance in Atlantic halibut (*Hippoglossus hippoglossu*s) larvae [16].

In larval culture, determining a particular nutrient requirement is critical; however, quantitative studies of the optimum nutrient requirements in microdiets for larval fish are still lacking [11]. Protein is the most expensive ingredient in fish feed and is usually included in large quantities to meet the demand needed for fish growth and body maintenance [17]. Dietary protein requirement of fish depends on the quantity and quality of amino acids (AA), fish size (age), water temperature, and other rearing conditions [17, 18]. It was suggested that the optimal protein requirement for juvenile (initial weight of 7.3 ± 0.04 g; means ± SD) rockfish should not be less than 48.6%, but not greater than 50% at the isoenergetic (16.7 kJ/g diet) level [19]. A similar study focusing on the protein requirement of rockfish at a much smaller size (initial weight of 3.2 ± 0.01 g; means ± SD) suggested the requirement of 50% [20]. Fish metamorphosis at different stages differ physiologically and have higher needs for nutrients at the larval stage than juvenile and growing stages [21].

To our knowledge, there are no previous studies on the protein requirement in microdiets for rockfish larval. In the light of the lack of study on rockfish larval nutrition, this study focuses on evaluating the protein requirement in granulated microdiets for larval rockfish and the growth performance of larvae fed with different granulated microdiets. Understanding this requirement will help to build more knowledge on the optimization of microdiets that could improve larval fish.

## 2. Materials and Methods

### 2.1. Spawning and Larvae Rearing Conditions

Female broodstocks in net cages, with fully distended abdomen appearing to be near parturition, were selected in Heaksando fishery (Shinan-gun, Jeollanam-do, Korea; 34°38′ 43.0^″^ N 125°24′ 42.2^″^ E) and transferred to Sinbi hatchery (Namhae-gun, Gyeongsangnam-do, Korea; 34°47′ 12.4^″^ N 128°03′ 07.0^″^ E). Then, broodstocks were held in a well-aerated, 20-ton fiberglass reinforced plastic (FRP) circular tank filled with seawater sterilized by ultraviolet radiation. After parturition, the females were removed, and the larvae were kept in the FRP tank for 9 days after parturition (DAP). During this period, water temperature ranged from 16.9 to 21.0°C (mean temperature± SD: 18.9 ± 0.71°C) in the tank. The feeding schedule and ration for the larvae are presented in Table 1. The larvae were fed with rotifers from 1 to 6 DAP and then fed with *Artemia* nauplii beginning from 6 to 10 DAP. Rotifer and *Artemia* nauplii were nutritionally enriched with S. Presso (Inve, Dendermonde, Belgium) at a dose recommended by the manufacturer before feeding. During the periods when feeding overlapped, two types of feed were supplied together. Granulated microdiets (#3 and #4) were fed to the larvae from 10 DAP to 29 DAP. The cessation of the experiment coincides with the early juvenile stage. The larvae were fed with the microdiets 8 to 12 times a day between 06 : 00 and 18 : 00 h.

### 2.2. Preparation of the Experimental Diets

The feeding composition of the experimental diets is shown in Table 2. Sardine meal, hydrolyzed fish soluble, krill meal, wheat gluten, and taurine were included as the main protein sources in the experimental diets, and fish oil was included as the main lipid sources. Additionally, alpha starch and dextrin were used as the main carbohydrate sources in the experimental diets. The five experimental microdiets were prepared and correspondingly named according to their crude protein content (4% increment) as the CP42, CP46, CP50, CP54, and CP58 diets at the expense of dextrin and fish oil while maintaining a constant gross energy level (18.4 kJ/g). The ingredients were thoroughly mixed and ground by an air Z-mill (SK Z-mill 0405, Seishin Enterprise Co. Ltd., Tokyo, Japan). The mixed ingredients were granulated with a granulator (Flow-Z granulator, Okawara Co. Ltd., Shizuoka, Japan) and then dried at 60°C by a dryer (Horizontal fluid bed dryer, Okawara Co. Ltd., Shizuoka, Japan). The granulated microdiets were sieved and prepared in two sizes [#3 (0.31–0.48 *μ*m), and #4 (0.48–0.63 *μ*m)]. The granulated microdiets were coated and packed. The efficacy of the experimentally formulated microdiets was compared with two types of overseas marketed granulated microdiets, Inve (IV) and LL (love larva) from Belgium and Japan, respectively, and a commercially available crumble diet in Korea. All experimental diets were fed to triplicate groups of larval fish.

### 2.3. Experimental Conditions

Seven thousand and two hundred larval fish (initial weight of 13.7 mg) were randomly distributed in 24, 70 L square plastic tanks (300 larvae per tank) after 9 DAP. The smaller size (#3) of the granulated microdiets was supplied at 10 DAP, ending at 27 DAP, while the larger size (#4) was supplied at 24 DAP, ending at the 29 DAP. 1 g of PRO-W (Inve, Dendermonde, Belgium) and MIC-F (Inve, Dendermonde, Belgium) was added to each tank daily to maintain water quality. The flow rate of sterilized seawater was 0.45 L/tank/min, and the residues accumulated on the bottom of the tank were daily siphoned and cleaned. At the end of the feeding trial on 29 DAP, fish were starved for 24 h.

### 2.4. Sampling Collection and Chemical Analysis

All surviving fish in each tank were counted, collectively weighed, and sampled for growth and nutritional analysis. Measurements were made after the fish were anesthetized with tricaine methanesulfonate (MS-222) at 100 ppm. Samples were stored at –70°C before being analyzed. After defrosting the sampled fish, 50 samples were randomly selected from each tank to measure the total length by an eyepiece micrometer (OM-500 N, NaRiKa, Tokyo, Japan) while being viewed under a microscope (Eclipse E200, Nikon, Tokyo, Japan). Weight gain was determined using an electronic balance (ATX224, Shimadzu Corporation, Kyoto, Japan). Before further examinations, the samples were homogenized prior to chemical analysis. The crude protein content was determined by the Kjeldahl method (Auto Kjeldahl System, Buchi B-324/435/412, Flawil, Switzerland), the crude lipid was determined using an ether extraction method, moisture was determined by drying in an oven at 105°C for 24 h, and ash content was determined by incineration using a muffle furnace at 550° C for 4 h. The procedure followed a standard method [22]. AA profiles of the experimental microdiets and fish's whole body were determined by using a high-speed AA analyzer (Hitachi L-8800, Tokyo, Japan) after sample hydrolysis in 6 N HCl for 24 h at 110°C.

### 2.5. Statistical Analysis

One-way analysis of variance (ANOVA) and Duncan's multiple range test [23] were applied to determine dietary treatment effect by using SPSS program version 19.0 (SPSS Michigan Avenue, Chicago, IL, USA). Broken line analysis [24] was used to determine dietary protein requirements for larval rockfish using the SAS version 9.3 program (SAS Institute, Cary, NC, USA). The percentage data were transformed to arcsine prior to statistical analysis.

## 3. Results

### 3.1. Profiles of AA of the Experimental Diets

The AA profiles of the experimental microdiets are presented in Table 3. An essential and nonessential AA increased as the protein content in the experimental microdiets increased.

### 3.2. Survival and Growth Performance of Rockfish Larvae

Survival, weight gain, and total length of rockfish larvae fed experimental diets are presented in Table 4. The survival ranged from 50.4 to 56.3% and was not significantly (*P* > 0.3) affected by the experimental diets. However, the weight gain (%) of rockfish larvae fed the CP54, IV, and LL diets was significantly (*P* > 0.0001) higher than that of fish fed the CP42, CP46, CP50, and CP58 diets. The total length of the rockfish larvae fed the IV and LL diets was significantly (*P* > 0.0001) longer than that of the fish larvae fed all other diets. The total length of the rockfish fed the CP54 diet was also significantly (*P* < 0.05) longer than that of larval fish fed all other formulated diets (CP42, CP46, CP50, and CP58). The shortest total length was obtained in larval fish fed the crumble diet.

### 3.3. Chemical Composition of the Whole Body of Rockfish Larvae

The chemical composition of the whole body of rockfish larvae at the end of the feeding trial is presented in Table 5. The moisture content of the whole fish body ranged from 80.2 to 80.6%, crude protein content ranged from 12.4 to 12.7%, and crude lipid ranged from 2.3 to 2.7%. These parameters were not significantly (*P* > 0.05) different between the experimental diets. However, the ash content of larval fish fed the CP46 diet was significantly (*P* < 0.01) higher than that of larval fish fed the CP42, CP50, CP54, CP58, and IV diets, but not significant (*P* > 0.05) different from that of larval fish fed the LL and crumble diets.

### 3.4. AA Profiles of the Whole Body and Protein Requirement of Larval Rockfish

**Figure 1:**
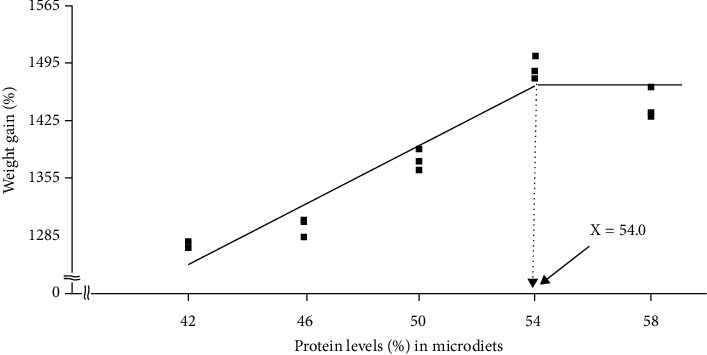
Effect of various protein levels in granulated microdiets on weight gain of rockfish larvae (means of triplicate ± SE). *Y* = *Y* = 1453.7–15.8 (*R*–*X*_LR_), *R* = 54.0 ± 1.71 (SE).

AA profiles of larval rockfish at the end of the feeding trial are shown in Table 6. The contents of alanine, glutamic acid, histidine, leucine, proline, and threonine showed significant differences (*P* < 0.05) among the experimental diets. A broken line analysis based on the weight gain (%) of larval rockfish at various protein levels in microdiets indicated that the protein requirement (*R*) for larval rockfish is estimated to be 54.0% [*Y* = 1453.7–15.8 (*R*–*X*_LR_), *R* = 54.0 ± 1.71 (SE)] (Figure 1).

## 4. Discussion

Rockfish growth from larval to the juvenile stage is typically approximately 24 mm in total length at 30 DAP [24]. This study was aimed at improving the growth of larval fish using granulated microdiets containing essential nutrients and reducing the prolonged time of feeding live feed by feeding them with microdiets at 10 DAP and terminating the study at 29 DAP to ensure only larval are used.

The development of microdiets has been suggested to be a positive solution to the substitution for live foods in larval nutrition [25, 26]. In this study, the administration of granulated microdiets to larval rockfish influenced the weight gain (%) and total length (mm). The higher greater weight and the longer total length of rockfish larvae are consistent with the findings of a study on sea bream (*Sparus aurata*) fed formulated microdiets with different levels of soybean lecithin [27] and in olive flounder larvae fed granulated microdiets containing various levels of protein [15]. The inclusion of microdiets also improved the growth performance of pike silverside (*Chirostoma estor*) as compared to a commercial diet [25].

Fish larvae have a relatively high protein requirement, but there is an optimal level of inclusion. The weight gain and total length of larval rockfish increased with increased dietary protein up to a level of 54%, but decreased with an increase in crude protein content. Improved growth performance with increased dietary protein levels to some extent is common in most fish, but an excessive inclusion in the diet may lead to reduced growth [19]. The high demand for energy for catabolism rather than protein deposition will result in a poor growth rate of fish when diets are excessively high in protein [28]. The decrease in growth as a result of high protein may be caused by the build-up of toxic nitrogen compounds, which can affect the growth of rockfish [29, 30]. Therefore, the protein requirement of rockfish larvae fed granulated microdiets was estimated to be 54.0% according to the broken line analysis of weight gain of larval fish. Previous studies have been done to ascertain dietary protein requirements (45–50%) for juvenile rockfish [19, 20]. Differences in protein requirements between this study (54%) and those of the previous studies (45-50%) may have occurred because of age differences (size) and because the nutrient requirements of larval fish differ from those of juvenile fish. Fish larval have higher dietary protein requirements than juvenile fish, and this has been corroborated by previous studies [15, 31, 32]. For instance, the protein requirement was estimated to be 55.4% when larval olive flounder were fed with granulated microdiets containing various levels (42–58%) in crude protein in a study [15]. However, the dietary protein requirements for juvenile (initial weight of 4.1 ± 0.02 g; means ± SD) and growing (initial weight of 13.3 ± 0.06 g; means ± SD) olive flounder were reported to be 46.4–51.2 and 40–44%, respectively [33], and decreased as fish grew. Both studies used different protein sources, which may affect the protein requirements of fish, and the metabolism of larval fish differs from that of juvenile ones when fed with various dietary proteins [34]. The optimal P/E (29.4 mg protein/kJ gross energy) in the CP54 diet in this study was within the range of the P/E ratio suggested in a previous study [19], in which optimum dietary P/E ranged from 21.5 to 35.4 mg protein/kJ for juvenile (initial weight of 7.3 ± 0.04 g; means ± SD) rockfish.

Amino acids absorbed from the feed are used for the effective synthesis of protein and for regulating the body's acid-base balance [35]. Limited study exists on the EAA requirements of rockfish larvae. The methionine requirement was estimated to be 1.37% for juvenile (initial weight of 43.6 ± 0.37 g; means ± SD) rockfish [36]. Poor growth performance in larval rockfish fed the CP42 and CP46 diets could be due to their lower methionine content compared to dietary methionine requirement reported [36]. Generally speaking, the smaller or younger fish required high nutrition requirements than the larger or older ones [3, 6]. The diets CP42 and CP46 and the crumble diets seemed to contain relatively low EAA content in most of EAA over the diets CP54 and CP58 and IV and LL diets in this study. Commercial aquafeeds often contain a relatively high protein content [37], and this may have facilitated the improved growth performance of larval rockfish observed in commercial microdiets commonly used in this study, such as the IV and LL diets. Larval gilthead bream fed with microdiets prepared using a modified microencapsulation procedure showed a similar survival rate (57%) in the 15-day feeding experiment [38].

The chemical composition of the fish's whole body is commonly ascertained by dietary nutrients [39, 40]. It was observed that moisture and crude protein and lipid content were not affected by the experimental diets in this study. This contrasts with the result of [20], in which the moisture, crude protein, and lipid contents of fish were affected by dietary protein and lipid contents. In another study [41], it was also reported that the crude protein and lipid content of the fish's whole body were affected by the content of dietary proteins. In both studies [20, 41], unlike this study, the ash content of fish was not affected by the dietary protein content. We observed no trend in ash content with dietary protein levels; however, fish fed the CP46 diet has the highest ash content. The protein to ash ratio of fish's body is not constant and can be altered through nutrition proteins and fish age [42].

A balanced AA is essential for fish growth, especially in larval fish [35, 43]. The AA profiles in the microdiets determine the dietary protein quality, and EAA must be adequately supplied to achieve improved growth of fish [10]. The EAA, such as histidine, leucine, and threonine content, and non-EAA, such as alanine, glutamic acid, and proline content of the fish's whole body differed among the experimental diets in this study. However, there is no visible trend in these differences. In a similar study involving the use of granulated microdiets on larval olive flounder, the AA profiles of the fish's whole body were not affected by protein levels in microdiets [15], and the reason for the observed differences in our study is unknown. This study could be helpful to develop microdiets for larval fish and replace the expensive imported commercial microdiets abroad.

## 5. Conclusions

Rockfish larvae fed the formulated microdiet containing 54% CP achieved comparable growth performance to fish fed imported (IV and LL) diets. Based on the broken line analysis of weight gain of rockfish larvae, protein requirement in microdiets was estimated to be 54.0%.

## Figures and Tables

**Table 1:** Feeding schedule showing ration, feeds, and frequency of feeding for rockfish larval from day 1 to 29 days after parturition.

Day after parturition (DAP)	Rotifer (number/mL)	*Artemia* nauplii (number/mL)	Amount of microdiet (#3) (g/time)	Amount of mixture of #3 and #4 microdiets at 1 : 1 (g/time)	Amount of microdiet (#4) (g/time)	Daily feeding frequency
1–5	12.00					
6	6.00	6.00				
7–9		12.00				
10		4.00	0.02			8.00
11–14			0.04			12.00
15–20			0.20			12.00
21–23			0.47			12.00
24–27				0.50		12.00
28–29					0.58	12.00

Size of #3 and #4 microdiets were 0.31–0.48 and 0.48–0.63 *μ*m, respectively.

**Table 2:** Feed ingredients of the experimental microdiets (%, dry matter basis).

	Experimental diets							
	CP42	CP46	CP50	CP54	CP58	IV	LL	Crumble
Ingredients (%)								
Sardine meal^a^	16.00	18.00	20.00	22.00	24.00	Closed		
Hydrolyzed fish soluble^b^	16.00	18.00	20.00	22.00	24.00			
Krill meal	27.00	29.00	31.00	33.00	35.00			
Wheat gluten	2.00	2.00	2.00	2.00	2.00			
Taurine	2.50	2.50	2.50	2.50	2.50			
*α*-starch	3.00	3.00	3.00	3.00	2.00			
Dextrin	21.00	15.50	10.00	4.50	0.00			
Fish oil	6.00	5.50	5.00	4.50	4.00			
Soybean lecithin	0.65	0.65	0.65	0.65	0.65			
Vitamin premix^c^	4.00	4.00	4.00	4.00	4.00			
Mineral premix^d^	1.00	1.00	1.00	1.00	1.00			
Choline chloride (50%)	0.85	0.85	0.85	0.85	0.85			
Nutrients (%)								
Dry matter	98.2	98.3	98.7	99.1	98.5	95.6	98.2	97.4
Crude protein	42.4	46.5	50.3	54.2	58.2	56.0	57.1	56.0
Crude lipid	14.0	14.4	14.5	14.7	15.1	10.4	14.7	8.9
Ash	8.8	8.5	8.6	8.5	8.5	8.5	12.2	11.0
Gross energy (kJ/g diet) ^e^	18.2	18.4	18.4	18.4	18.5	17.5	17.8	16.8
P/E (mg protein/kJ)	23.2	25.3	27.4	29.4	31.4	31.9	32.0	33.3

^a^Sardine meal was imported from Chile. ^b^Hydrolyzed fish soluble was imported from France. ^c^Vitamin premix contained the following amount which were diluted in brewer's yeast (mg kg/diet): L-ascorbic acid, 51.24; DL-*α*-tocopheryl acetate, 150.0; thiamin hydrochloride, 20.0; riboflavin, 40.0; pyridoxine hydrochloride, 20.0; nicotinic acid, 150.0; D-calcium-pantothenate, 70.0; inositol, 300.0; D-biotin, 0.2; folic acid, 10.0; p-aminobenzoic acid, 18.2; menadione sodium hydrogen sulfite, 10.0; retinyl acetate, 6.0; cyanocobalamin, 0.001. ^d^Mineral premix contained the following amount which were diluted in brewer's yeast (mg kg/diet): MgSO_4_·7H_2_O, 496.92; C_4_H_2_FeO_4_, 65.8; FeSO_4_, 103.04; CuSO_4_, 5.97; CoSO_4_.7H_2_O, 3.42; CaI_2_, 3.91; ZnSO_4_, 68.85; Al (OH)_3_, 3.81; MnSO_4_·H_2_O, 65.8. ^e^Gross energy is calculated based on 16.7 kJ/g for protein and carbohydrate and 37.7 kJ/g for lipid (Garling and Wilson, 1976).

**Table 3:** Amino acid (AA) profiles of the main protein sources and experimental diets (% of dietary protein).

	Sardine meal	Hydrolyzed fish soluble	Krill meal	Experimental diets							
				CP42	CP46	CP50	CP54	CP58	IV	LL	Crumble
*Essential AA*											
Arginine	6.36	6.04	5.86	6.25	6.04	5.96	5.90	5.88	6.14	6.69	5.25
Histidine	2.27	1.90	1.98	2.24	2.22	2.25	2.23	2.20	2.34	3.22	2.29
Isoleucine	4.90	3.41	5.12	4.74	4.56	4.53	4.46	4.43	4.66	5.01	3.41
Leucine	8.23	5.81	7.84	7.71	7.46	7.44	7.29	7.27	7.54	8.34	6.09
Lysine	8.78	6.53	7.12	7.33	7.18	7.16	7.14	7.11	7.41	8.49	5.91
Methionine	3.53	2.66	2.77	2.76	2.62	2.70	2.71	2.75	3.04	3.05	2.54
Phenylalanine	4.99	3.51	4.44	4.29	4.13	4.12	4.06	3.99	4.34	4.48	3.34
Threonine	4.63	3.74	4.23	4.39	4.24	4.19	4.15	4.07	4.36	4.80	3.50
Valine	5.72	4.31	4.96	5.00	4.86	4.85	4.80	4.74	4.88	5.69	4.14
*Nonessential AA*											
Alanine	6.20	6.25	5.19	5.94	5.81	5.81	5.76	5.76	5.38	6.53	5.39
Aspartic acid	10.69	8.28	10.12	9.76	9.42	9.30	9.21	9.07	9.88	10.47	7.57
Cysteine	1.22	0.81	0.68	0.94	0.90	0.99	0.94	1.01	0.88	1.07	0.80
Glutamic acid	13.76	11.60	12.67	14.55	13.94	13.72	13.47	13.11	14.48	14.34	12.13
Glycine	5.20	9.47	4.32	6.39	6.15	6.14	6.11	6.12	5.02	6.46	6.34
Proline	3.59	5.06	3.61	4.29	4.13	4.12	4.06	3.99	4.20	4.47	4.61
Serine	4.53	4.11	3.84	4.17	4.02	3.96	3.91	3.87	4.23	4.43	3.43
Tyrosine	3.43	2.27	2.12	3.84	3.66	3.64	3.56	3.52	4.05	3.20	2.29

**Table 4:** Survival (%), weight gain (%), and total length (mm) of rockfish larvae (initial weight of 13.7 mg) fed the experimental diets at the end of the 20-day feeding trial.

Experimental diets	Final weight (mg/fish)	Survival (%)	Weight gain (%)^1^	Total length (mm)
CP42	187.8 ± 0.33^d^	54.3 ± 2.65	1273.8 ± 2.41^d^	22.2 ± 0.01^d^
CP46	190.9 ± 0.88^d^	54.6 ± 1.56	1296.7 ± 6.47^d^	22.2 ± 0.00^d^
CP50	201.9 ± 1.00^c^	54.9 ± 1.82	1377.3 ± 7.34^c^	22.2 ± 0.01^d^
CP54	217.2 ± 1.07^a^	55.2 ± 0.91	1488.9 ± 7.86^a^	22.5 ± 0.00^b^
CP58	211.0 ± 1.57^b^	55.2 ± 1.13	1443.7 ± 11.51^b^	22.3 ± 0.01^c^
IV	218.3 ± 0.79^a^	53.4 ± 0.91	1497.1 ± 5.76^a^	22.6 ± 0.01^a^
LL	219.7 ± 0.71^a^	56.3 ± 0.88	1507.6 ± 5.17^a^	22.6 ± 0.01^a^
Crumble	184.1 ± 1.57^e^	50.4 ± 2.04	1247.1 ± 11.50^e^	22.1 ± 0.01^e^
*P* value	*P* < 0.0001	*P* > 0.3	*P* < 0.0001	*P* < 0.0001

Values (means of triplicate± SE) in the same column sharing the same superscript letter are not significantly different (*P* > 0.05). ^1^Weight gain (%) = (Final weight of fish–initial weight of fish) × 100/initial weight of fish.

**Table 5:** Proximate composition (% of wet weight) of the whole body of rockfish larvae fed the experimental diets at the end of the feeding trial.

Experimental diets	Moisture	Crude protein	Crude lipid	Ash
CP42	80.4 ± 0.10	12.4 ± 0.12	2.4 ± 0.10	3.0 ± 0.02^c^
CP46	80.3 ± 0.20	12.5 ± 0.09	2.5 ± 0.09	3.2 ± 0.08^a^
CP50	80.5 ± 0.15	12.5 ± 0.08	2.5 ± 0.11	3.0 ± 0.05^bc^
CP54	80.3 ± 0.12	12.5 ± 0.12	2.5 ± 0.06	2.9 ± 0.02^c^
CP58	80.2 ± 0.20	12.6 ± 0.09	2.5 ± 0.13	3.0 ± 0.06^c^
IV	80.5 ± 0.22	12.6 ± 0.05	2.7 ± 0.06	2.9 ± 0.07^c^
LL	80.5 ± 0.23	12.7 ± 0.04	2.7 ± 0.04	3.1 ± 0.05^ab^
Crumble	80.6 ± 0.02	12.6 ± 0.03	2.4 ± 0.08	3.1 ± 0.03^ab^
*P* value	*P* > 0.6	*P* > 0.4	*P* > 0.07	*P* < 0.01

Values (means of triplicate ±SE) in the same column sharing the same superscript letter are not significantly different (*P* > 0.05).

**Table 6:** Amino acid (AA) profiles of the whole body of rockfish larvae fed the experimental diets at the end of the feeding trial (% of wet weight).

	Experimental diets								*P* value
	CP42	CP46	CP50	CP54	CP58	IV	LL	Crumble	
*Essential AA*									
Arginine	0.73 ± 0.025	0.79 ± 0.042	0.71 ± 0.021	0.71 ± 0.023	0.67 ± 0.040	0.71 ± 0.036	0.68 ± 0.055	0.74 ± 0.009	*P* > 0.4
Histidine	0.27 ± 0.012^abc^	0.29 ± 0.006^a^	0.25 ± 0.006^c^	0.26 ± 0.003^bc^	0.26 ± 0.010^bc^	0.27 ± 0.009^abc^	0.28 ± 0.009^ab^	0.28 ± 0.007^abc^	*P* < 0.04
Isoleucine	0.48 ± 0.020	0.51 ± 0.025	0.47 ± 0.006	0.47 ± 0.010	0.52 ± 0.088	0.52 ± 0.012	0.55 ± 0.017	0.52 ± 0.017	*P* > 0.7
Leucine	0.82 ± 0.035^bc^	0.87 ± 0.053^ab^	0.81 ± 0.013^bc^	0.81 ± 0.019^bc^	0.77 ± 0.021^c^	0.91 ± 0.015^ab^	0.96 ± 0.030^a^	0.91 ± 0.033^ab^	*P* < 0.01
Lysine	0.74 ± 0.025	0.74 ± 0.032	0.74 ± 0.015	0.72 ± 0.007	0.71 ± 0.036	0.73 ± 0.013	0.71 ± 0.027	0.74 ± 0.043	*P* > 0.7
Methionine	0.34 ± 0.018	0.34 ± 0.015	0.33 ± 0.007	0.34 ± 0.012	0.34 ± 0.012	0.35 ± 0.003	0.37 ± 0.009	0.35 ± 0.012	*P* > 0.3
Phenylalanine	0.47 ± 0.015	0.46 ± 0.009	0.46 ± 0.000	0.45 ± 0.009	0.45 ± 0.017	0.47 ± 0.006	0.50 ± 0.015	0.47 ± 0.019	*P* > 0.1
Threonine	0.55 ± 0.019^bcd^	0.59 ± 0.026^ab^	0.54 ± 0.003^cd^	0.54 ± 0.007^cd^	0.53 ± 0.015^d^	0.59 ± 0.012^abc^	0.61 ± 0.019^a^	0.58 ± 0.012^abcd^	*P* < 0.02
Valine	0.58 ± 0.023	0.58 ± 0.007	0.57 ± 0.006	0.57 ± 0.015	0.56 ± 0.018	0.60 ± 0.015	0.64 ± 0.022	0.61 ± 0.017	*P* > 0.09
*Nonessential AA*									
Alanine	0.71 ± 0.030^ab^	0.75 ± 0.046^abc^	0.71 ± 0.017^ab^	0.70 ± 0.017^c^	0.67 ± 0.021^c^	0.79 ± 0.015^ab^	0.83 ± 0.034^a^	0.79 ± 0.028^ab^	*P* < 0.01
Aspartic acid	1.13 ± 0.042	1.12 ± 0.012	1.10 ± 0.019	1.09 ± 0.024	1.07 ± 0.042	1.15 ± 0.023	1.17 ± 0.043	1.14 ± 0.025	*P* > 0.1
Cysteine	0.13 ± 0.009	0.14 ± 0.003	0.13 ± 0.000	0.17 ± 0.044	0.12 ± 0.003	0.14 ± 0.003	0.14 ± 0.003	0.13 ± 0.006	*P* > 0.5
Glutamic acid	1.55 ± 0.059^bc^	1.67 ± 0.085^ab^	1.53 ± 0.012^bc^	1.51 ± 0.032^bc^	1.48 ± 0.054^c^	1.67 ± 0.040^ab^	1.72 ± 0.043^a^	1.64 ± 0.031^ab^	*P* < 0.03
Glycine	0.76 ± 0.022	0.76 ± 0.020	0.76 ± 0.010	0.75 ± 0.023	0.74 ± 0.030	0.82 ± 0.020	0.86 ± 0.039	0.83 ± 0.027	*P* > 0.06
Proline	0.51 ± 0.009^b^	0.54 ± 0.03^ab^	0.50 ± 0.012^b^	0.50 ± 0.017^b^	0.48 ± 0.019^b^	0.57 ± 0.010^a^	0.59 ± 0.017^a^	0.58 ± 0.020^a^	*P* < 0.003
Serine	0.55 ± 0.017	0.55 ± 0.006	0.55 ± 0.003	0.54 ± 0.012	0.53 ± 0.022	0.57 ± 0.012	0.56 ± 0.038	0.56 ± 0.012	*P* > 0.3
Tyrosine	0.34 ± 0.012	0.41 ± 0.050	0.36 ± 0.026	0.37 ± 0.020	0.33 ± 0.007	0.34 ± 0.009	0.35 ± 0.009	0.35 ± 0.013	*P* > 0.3

Values (means of triplicate ± SE) in the same row sharing the same superscript letter are not significantly different (*P* > 0.05).

## Data Availability

The data are available on request from the authors.
